# Monkeypox Disease Transmission in an Experimental Setting: Prairie Dog Animal Model

**DOI:** 10.1371/journal.pone.0028295

**Published:** 2011-12-02

**Authors:** Christina L. Hutson, Darin S. Carroll, Nadia Gallardo-Romero, Sonja Weiss, Cody Clemmons, Christine M. Hughes, Johanna S. Salzer, Victoria A. Olson, Jason Abel, Kevin L. Karem, Inger K. Damon

**Affiliations:** 1 Poxvirus and Rabies Branch, Division of High-Consequence Pathogens and Pathology, National Center for Emerging and Zoonotic Disease, Centers for Disease Control and Prevention, Atlanta, Georgia, United States of America; 2 Program in Population Biology, Ecology and Evolution, Emory University, Atlanta, Georgia, United States of America; Blood Systems Research Institute, United States of America

## Abstract

Monkeypox virus (MPXV) is considered the most significant human public health threat in the genus *Orthopoxvirus* since the eradication of variola virus (the causative agent of smallpox). MPXV is a zoonotic agent endemic to forested areas of Central and Western Africa. In 2003, MPXV caused an outbreak in the United States due to the importation of infected African rodents, and subsequent sequential infection of North American prairie dogs (*Cynomys ludovicianus*) and humans. In previous studies, the prairie dog MPXV model has successfully shown to be very useful for understanding MPXV since the model emulates key characteristics of human monkeypox disease. In humans, percutaneous exposure to animals has been documented but the primary method of human-to-human MPXV transmission is postulated to be by respiratory route. Only a few animal model studies of MPXV transmission have been reported. Herein, we show that MPXV infected prairie dogs are able to transmit the virus to naive animals through multiple transmission routes. All secondarily exposed animals were infected with MPXV during the course of the study. Notably, animals secondarily exposed appeared to manifest more severe disease; however, the disease course was very similar to those of experimentally challenged animals including inappetence leading to weight loss, development of lesions, production of orthopoxvirus antibodies and shedding of similar levels or in some instances higher levels of MPXV from the oral cavity. Disease was transmitted via exposure to contaminated bedding, co-housing, or respiratory secretions/nasal mucous (we could not definitively say that transmission occurred via respiratory route exclusively). Future use of the model will allow us to evaluate infection control measures, vaccines and antiviral strategies to decrease disease transmission.

## Introduction

Monkeypox virus (MPXV) and variola virus (the causative agent of smallpox) are members of the genus *Orthopoxvirus*. While smallpox has been eradicated from the human population and viral isolates only remain in secure Biosafety Level 4 laboratories; MPXV is a zoonotic pathogen endemic to Central and Western Africa where it can cause human infection and even mortality, and is maintained in the wild by undetermined rodent reservoir(s) [1]–[3]. In 2003 MPXV caused the first outbreak of human disease outside of Africa within the United States [4]. The virus was introduced due to importation of infected rodents; human disease resulted from the subsequent infection of North American black-tailed prairie dogs (*Cynomys ludovicianus)* which in turn efficiently transmitted disease to humans [5], [6]. Anecdotally, it appeared prairie dogs transmitted MPXV within households, pet store, or other settings. Previous studies have defined two distinct MPXV clades, West African and Congo Basin [7], [8]; the U.S. outbreak was due to an importation of the West African clade MPXV. In humans, West African MPXV causes a milder disease, <1% mortality and is rarely associated with person to person transmission [9], [10]. However, Congo Basin MPXV causes approximately 10% mortality and human to human transmission has been observed; up to six sequential interhuman transmission events have been laboratory-documented [11]. Both MPXV and smallpox are believed to be transmitted between humans primarily via respiratory secretions.

As evidenced by the 2003 US outbreak, as well as the ongoing outbreaks of MPXV within Africa, there is a continued need to study and understand the details of viral transmission among hosts. Additionally, MPXV provides a surrogate for the study of related orthopoxviruses including variola virus. An ideal animal model is one that would emulate key features of human disease including: utilizing a route of infection that mimics the natural transmission of the pathogen in humans; the ability to obtain disease with an infectious dose equivalent to that causing disease in humans; as well as having a disease course, morbidity and mortality similar to what is observed with human disease. Previous studies of the prairie dog MPXV model showed after intranasal or scarification challenge with a reasonable challenge dose, animals developed disease that closely resembled human monkeypox, including a protracted incubation period before the development of generalized lesions [12], [13]. Additionally, these studies have shown that the prairie dog model is valuable for the comparison of disease attributable to the two MPXV clades, primarily by differences in mortality and level of morbidity.

Thus far, only two published studies have experimentally investigated MPXV transmission; these studies have used baboons and tropical squirrels [14], [15]. Although infected animals were able to transmit the virus to naive animals in these studies, baboons are not practical to use in laboratory studies and the tropical squirrels were highly susceptible to MPXV without key features of human disease including the development of disseminated lesions. Herein we show that prairie dogs are able to transmit the virus to naive prairie dogs. Four primary challenged animals were inoculated via intranasal route with 9×10^3^ pfu (0.07XLD_50_) of West African MPXV and subsequently placed into one of three groups designed to evaluate fomites, contact and respiratory routes of disease transmission. This challenge inoculum was chosen because previous studies with a similar dosage resulted in low mortality and the development of morbidity including disseminated lesions as well as viral shedding [13].

In the current study, we sought to explore the hypothesis that infected prairie dogs are able to transmit the virus to naive animals. These studies would allow us to better understand the transmission events inferred through epidemiologic studies during the 2003 outbreak, and would allow us to better define the potential significance of respiratory routes of infection. The experimental groups that were utilized ascertained the ability of MPXV to be transmitted via fomite, direct contact, and respiratory secretions.

## Results

### Bedding/Fomite Group

**Figure 1:**
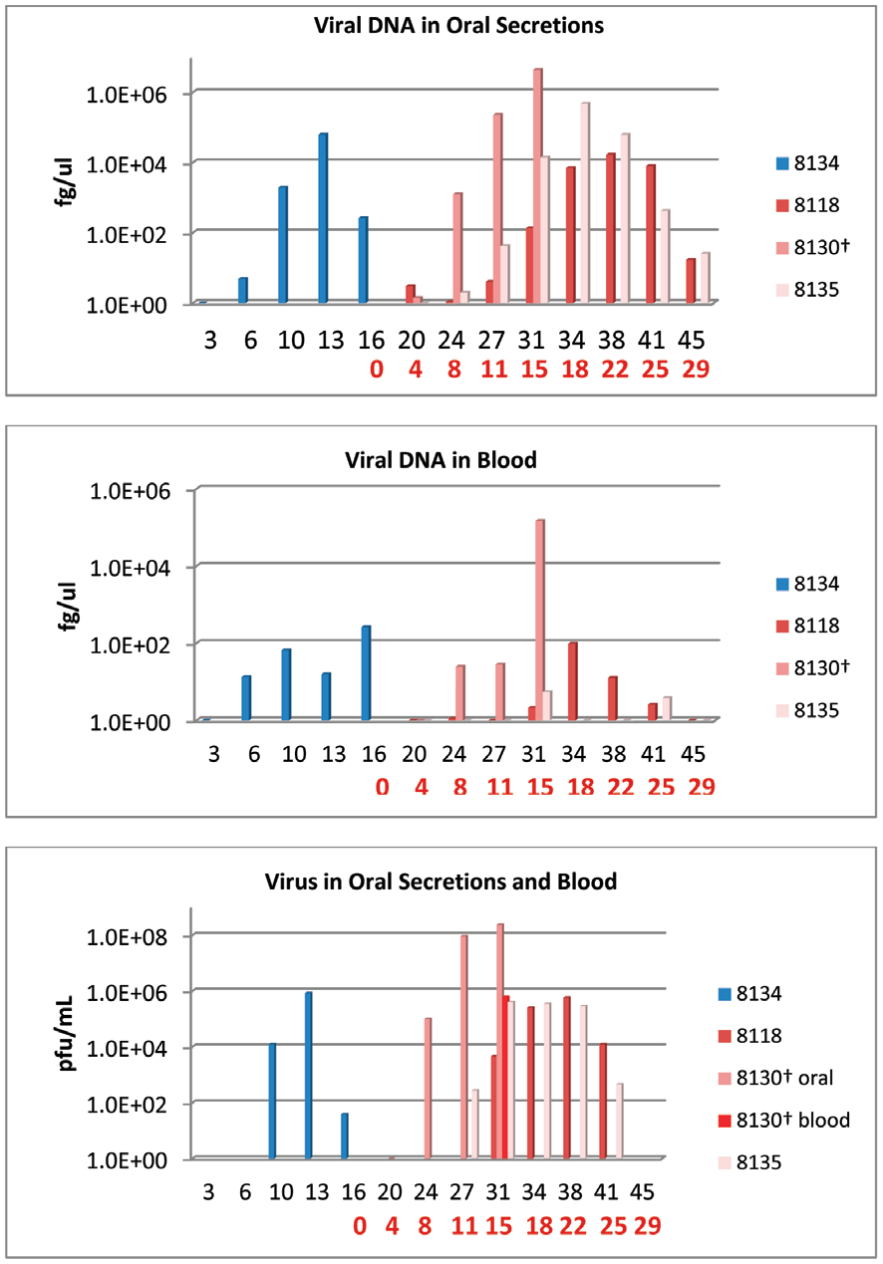
Bedding/fomite group: detection of viral DNA and viable virus from oropharyngeal swabs and blood. Four primary challenged animals were inoculated via intranasal route with 9×10^3^ pfu (0.07XLD_50_) of West African MPXV and placed into one of three experimental groups. For the bedding group, the primary challenged animal (blue bars) was housed for 16 days in a large trough, at which time the challenged animal was removed and three naive animals (red/mauve bars) were placed into the trough for housing. Blood and oral swabs were taken from each animal throughout the study and subsequently tested for viral DNA and live virus. Days are plotted on the X axis in black for day p.i. of primary challenged animal; in red for day post placement in trough. Samples yielding positive DNA (fg/ul) or viable virus (pfu/mL) are plotted on the Y axis in a log scale.

For the bedding/fomite group, one animal was challenged with virus and housed for 16 days in a large trough. After day 6 the bedding was not changed and the challenged animal was left in the trough until day 16 at which time it was removed and humanely euthanized. Three naive animals were then placed into the large trough for housing with the contaminated bedding and this bedding was not changed for 39 days. The primary animal (#8134) experimentally challenged i.n. with MPXV in the bedding group developed inappetence and labored breathing on day nine post-infection (p.i.). Infectious virus was initially demonstrated in oral cavity samples on day 10 (Fig. 1, Table 1). In prior studies, virus was detected in oral samples, on day 6 or 10 with an equivalent challenge dose [13]; in another study with a slightly higher challenge inoculum, viral shedding from oral, nasal, ocular and fecal samples began on or before day 12 [12]. By day 13, six MPXV pustules were present on the inner legs and abdomen of this animal. On day 16, this animal was removed from the trough so that three naive animals could be subsequently housed in the contaminated trough. At this time, #8134 manifest 11 pustules, most of which were forming crusts. As inappetence had subsided, lesions were crusting, and it was recovering from infection, this animal was humanely euthanized on day 16. Testing of serum samples demonstrated ELISA-measurable orthopoxvirus antibodies beginning on day 13 p.i. (Table 1). Blood and oral samples were positive for viral DNA beginning on day six; infectious viral shedding from the oral samples was characterized initially on day 10 (Fig. 1, Table 1). Blood from this experimentally infected animal did not yield viable virus. Weight loss (greater than 5%) began on day 10 p.i. for this animal and progressed to 10% of body weight by day 16 (Table 1).

**Table 1:** Disease/molecular findings in primary MPXV challenged prairie dogs (shaded) and secondarily infected prairie dogs.

Group	Bedding				Co-housed				Respiratory Secretions			
**Animal Number**	**8134**	8118	8130†	8135	**8127**	8133†	8136	8154	**8103**	8090	**8132**	8128†
**Day of Lesion Onset**	**13**	34(18)	27 (11)	34(18)	**13**	24	20	24	**13**	24	**10**	24
**Maximum Weight Loss**	**10%**	14%	12%	8%	**9%**	10%	1%	10%	**10%**	15%	**10%**	9%
**Viral DNA in Blood** **(days)**	**6->16**	31–41(15–25)	24–31(8–15)	31–41(15–25)	**6–24**	17–27	17–31	17–27	**6–24**	17–34	**6–27**	20–24
**Virus in Oral Secretions** **(days)**	**10->16**	31–41(15–25)	24–31(8–15)	27–41(11–25)	**10–27**	13–27	13–31	13–20	**6–17**	20–27	**6–17**	20–24
**Day of Initial Antibody Detection**	**13**	38(22)	31 (15)	38(22)	**13**	27	24	24	**13**	24	**13**	24

Four primary challenged animals (highlighted in grey) were inoculated via intranasal route with 9×10^3^ pfu (0.07XLD_50_) of West African MPXV and placed into one of three experimental groups with naive animals. The time-line shown is for day post infection of the primary challenged animal; for the naive bedding group numbers in parentheses are days post placement in the trough. Two times a week animals were anesthetized for sample collection, lesion count and weight measurements. Three of the naive animals (indicated by crosses) were euthanized due to extreme morbidity during the study.

Of the three naive, secondarily exposed animals, one manifest disease which presented earlier and progressed more rapidly than the other two animals in this group. Animal #8130 in the bedding group developed 14 lesions 11 days after placement in the trough. This animal was euthanized after 15 days in the contaminated trough due to extreme morbidity (including diarrhea and extensive oral lesions). The other two naives (#8118 and #8135) developed lesions 18 days after placement in the trough. Each of these animals developed a total of approximately 10 lesions. Orthopoxvirus antibodies were first detected from naive animal #8130 15 days after placement in the trough; and22 days from the other two naive animals (Table 1). Viral DNA from oral cavity samples collected from the naive animals were first detected four and eight days after placement in the trough; viral DNA in the blood was first detected on days eight (#8130) and 15 days after placement in the trough (Fig. 1, Table 1). Although all PCR positive blood samples taken throughout the course of this study were evaluated for viable virus, virus could be titrated from only two blood samples. One of these samples was from animal #8130 at time of euthanasia (Fig. 1). Viral shedding in oral secretions from each of the naive animals began on day eight (#8130), 11, and 15 post placement in the trough (Fig. 1, Table 1). Naive animal #8130 had a sharp drop in weight (>10% loss) occur between days 11–15; the other two naives had lost >5% of their starting weight by days 18 and 22 (Table 1). 39 days after placement in the trough, naive animals #8118 and #8135 had fully recovered from infection. No evidence of bites or scratches on any of these co-housed naive animals was observed during the course of infection or at time of necropsy.

### Co-Housed Group

**Figure 2:**
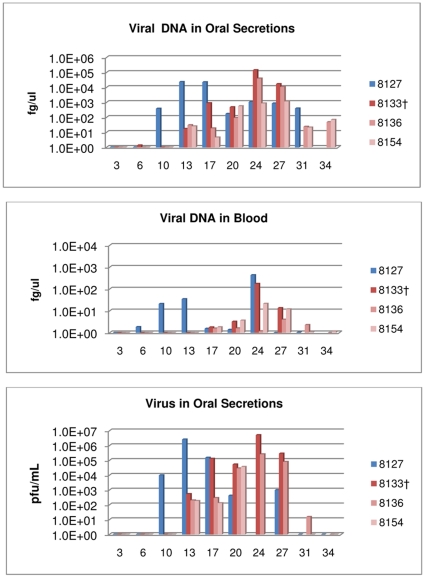
Co-house group: detection of viral DNA and viable virus from oropharyngeal swabs and blood. Four primary challenged animals were inoculated via intranasal route with 9×10^3^ pfu (0.07XLD_50_) of West African MPXV and placed into one of three experimental groups. For the co-housed group; primary challenged animal was challenged with virus and housed in a large trough with 3 naive animals. Blood and oral swabs were taken from each animal throughout the study and subsequently tested for viral DNA and live virus. Days are plotted on the X axis. Samples yielding positive DNA (fg/ul) or viable virus (pfu/mL) are plotted on the Y axis in a log scale.

For the co-housed portion of the study, one animal was challenged with virus and housed in a large trough with three naive animals for 38 days; bedding was changed weekly. After intranasal infection with MPXV, primary challenged animal (#8127) was co-housed with animal #s 8133, 8136 and 8154 approximately 15 minutes p.i. The primary challenged prairie dog had a typical MPXV disease presentation, similar to the primary challenged animal in the bedding group. Animal 8127 developed four lesions by day 13 p.i. During the course of infection the animal developed a total of approximately 15 lesions and had moderate weight loss (<10%) (Table 1). Orthopoxvirus antibodies were first detected on day 13 p.i. (Table 1). Viral DNA from blood samples was detected beginning on day six; viral DNA and viral shedding from oral cavity samples began on day 10 (Fig. 2). This primary challenged animal fully recovered from infection.

One naive animal (#8136) developed lesions 20 days p.i. of the primary challenged animal; the other two naive animals by day 24 p.i. (#s 8154 and 8133). Orthopoxvirus antibodies were first detected on days 24 (#s 8136 and 8154) and day 27 (#8133) (Table 1). Shedding of viral DNA and viable virus from oral cavity samples of all three secondarily infected animals began on day 13; viral DNA from blood samples was positive beginning on day 17 for all three animals (Fig. 2, Table 1). Two naive animals began losing weight on days 24 (#8133) and 27 (#8154); one naive never lost weight (#8136) (Table 1). On day 27, naive animal #8133 had developed too many lesions to count and due to extreme morbidity (diarrhea and number of lesions), this animal was humanely euthanized. Naive animals #8136 and #8154 survived MPXV infection. No evidence of bites or scratches on any of the co-housed animals was observed during the course of infection or at time of necropsy.

### Respiratory Secretion Groups

Four large metal rabbit cages with holes on one side were utilized to individually house four animals (two challenged, 2 naive) for the arm of the study designed to look at transmission via respiratory secretion. Cages were placed approximately one inch apart with ventilation holes from the challenged animals' cages facing the holed-side from the naive animals' cages within a Duo-Flow biosafety cabinet with negative directional airflow. Through this portion of the study, we were attempting to ascertain the ability of MPXV to be transmitted via respiratory route. However, because the cages were not separated by physical barrier, the question did arise as to whether these animals were able to touch noses through the holes in the cages and therefore exchange nasal discharge. If this scenario did occur, this would not be a true test of respiratory transmission but possibly a combination of respiratory transmission and/or nasal mucous secretion transmission.

#### Group 1 (Animals #8103 and #8090)

**Figure 3:**
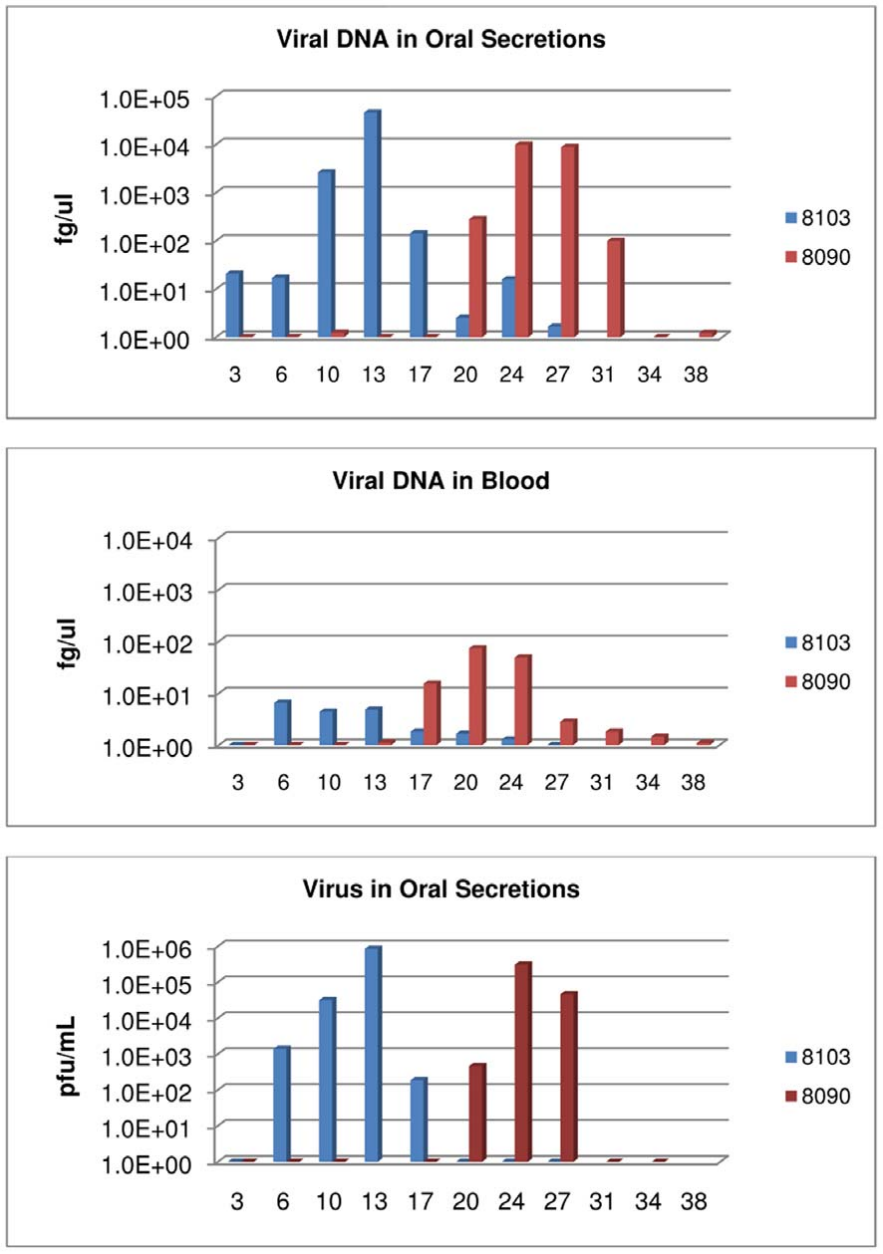
Respiratory secretions group-1: detection of viral DNA and viable virus from oropharyngeal swabs and blood. Four primary challenged animals were inoculated via intranasal route with 9×10^3^ pfu (0.07XLD_50_) of West African MPXV and placed into one of three experimental groups. For the respiratory secretion group; large metal rabbit cages with holes on one side were utilized to single house animals. Cages were placed approximately one inch apart with holes from the challenged animals' cages facing the holed-side from the naive animals' cages. Blood and oral swabs were taken from each animal throughout the study and subsequently tested for viral DNA and live virus. Days are plotted on the X axis. Samples yielding positive DNA (fg/ul) or viable virus (pfu/mL) are plotted on the Y axis in a log scale.

**Figure 4:**
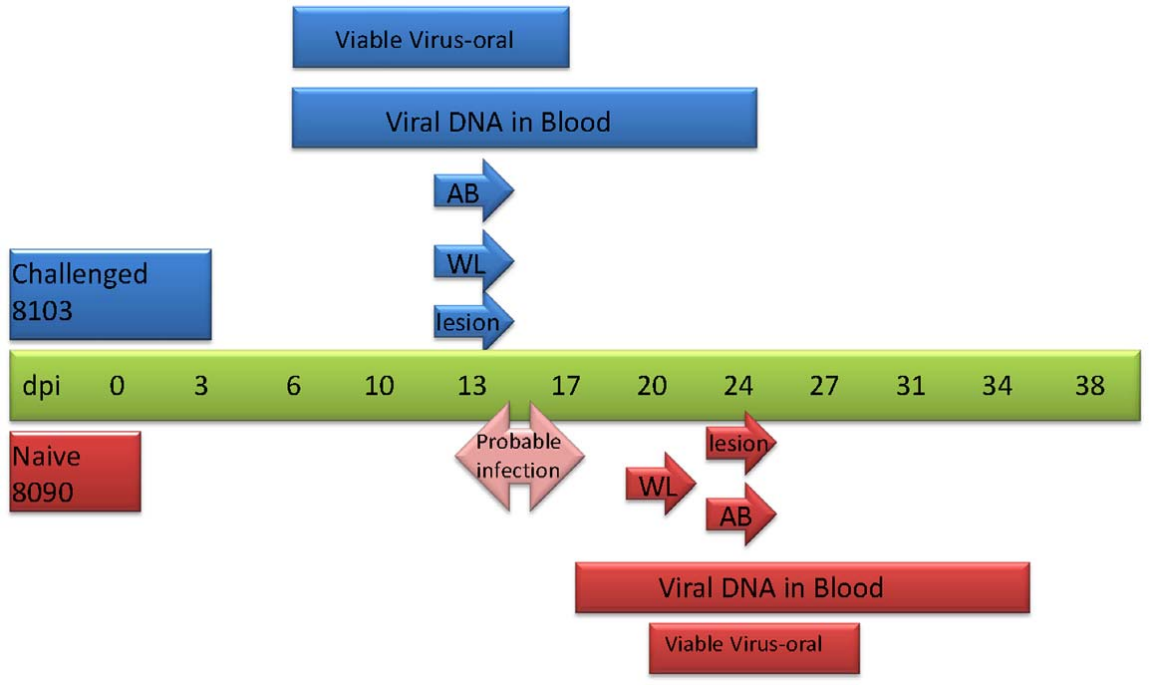
Respiratory secretions group 1 disease time-line. Primary challenged animal 8103 was inoculated via intranasal route with 9×10^3^ pfu (0.07XLD_50_) of West African MPXV and placed in a large metal cage with holes on one side which faced the holed-side of a cage housing naive animal 8090. Blood , oral swabs, weights and lesion counts were taken from each animal throughout the study. Total time that viable virus and viral DNA were present are illustrated with bars. The initial detection of antibodies, weight loss occurrence and lesion formation are illustrated with arrows. AB: development of antibodies. WL: weight loss >5%.

Primary challenged animal #8103 developed lesions by day 13 p.i.; a maximum of 10 lesions was counted on this animal at the peak of infection. Weight loss began on day 13 and reached a maximum of 10% on day 27 (Table 1). Orthopoxvirus antibodies were first detected on day 13 p.i. (Table 1). Oral samples from this animal yielded viral DNA on day three, viral shedding from this animal began on day six (Fig. 3, Table 1). Viral DNA in blood from this animal was detected on day six (Fig. 3, Table 1). This primary challenged animal (#8103) survived infection. Animal #8090 was the naive animal housed across from #8103. 20 days after MPXV challenge of #8103, animal #8090 developed an eye infection. A swab of the ocular surface yielded viral DNA and viable virus (data not shown). 24 days p.i. of the primary challenged animal, the naive animal #8090 had five lesions on the abdomen and legs. This animal developed a total of 10 lesions; weight loss for animal #8090 began on day 20 of the study and reached a maximum of 15% by day 27 (Table 1) and detection of orthopoxvirus antibodies occurred beginning on day 24 (Table 1). Viral DNA in the blood from #8090 was detected beginning on day 17; shedding of viral DNA and viable virus from oral cavity samples began on day 20 (Fig. 3, Table 1). Naive animal #8090 survived MPXV infection. The disease course for these two animals (experimentally challenged and secondarily infected) is illustrated in Figure 4 and shows that the secondarily infected animal's disease onset is delayed compared to the experimentally challenged animal, but otherwise the disease progression is very similar.

#### Group 2 (Animals #8132 and #8128)

**Figure 5:**
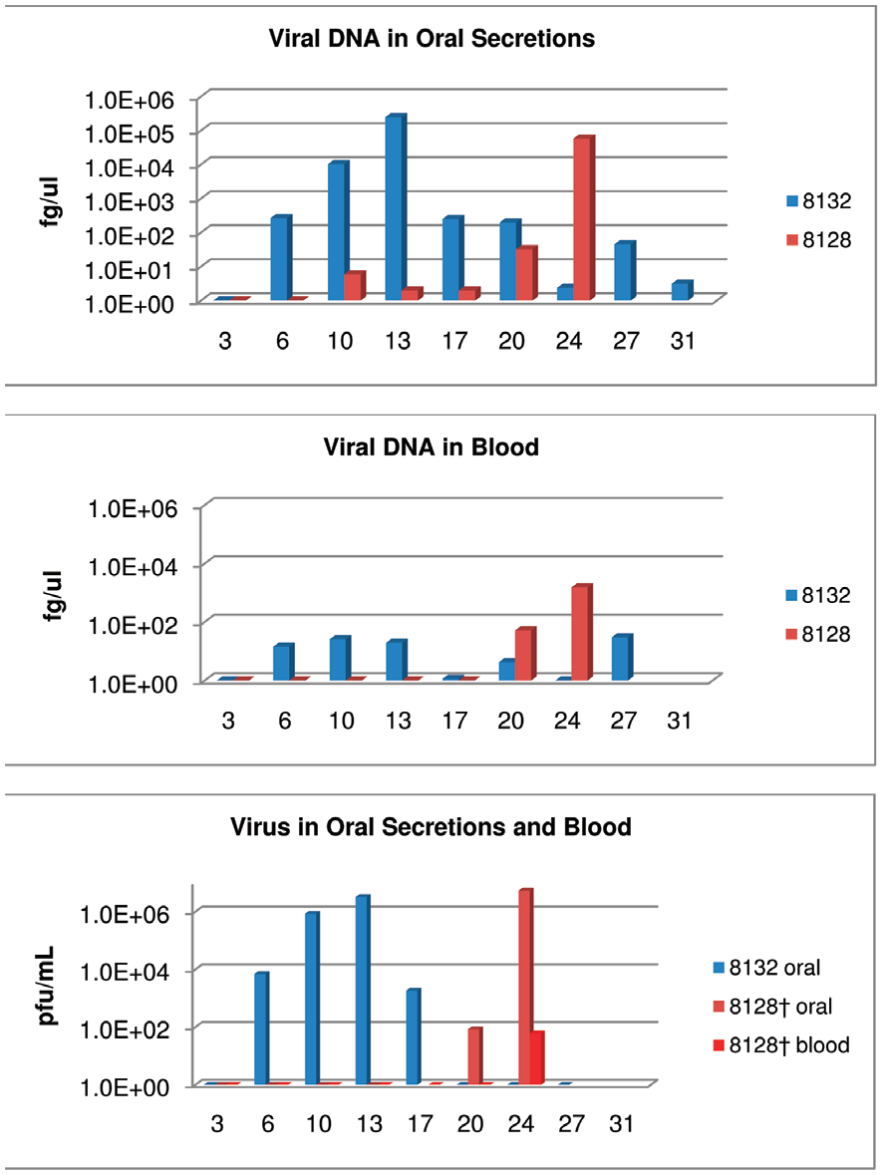
Respiratory secretions group-2: detection of viral DNA and viable virus from oropharyngeal swabs and blood. Four primary challenged animals were inoculated via intranasal route with 9×10^3^ pfu (0.07XLD_50_) of West African MPXV and placed into one of three experimental groups. For the respiratory secretion group; large metal rabbit cages with holes on one side were utilized to single house animals . Cages were placed approximately one inch apart with holes from the challenged animals' cages facing the holed-side from the naive animals' cages. Blood and oral swabs were taken from each animal throughout the study and subsequently tested for viral DNA and live virus. Days are plotted on the X axis. Samples yielding positive DNA (fg/ul) or viable virus (pfu/mL) are plotted on the Y axis in a log scale.

Primary challenged animal #8132 was challenged with MPXV and developed nine lesions by day 10 p.i. At the peak of infection, approximately 30 lesions developed on this animal. Weight loss began on day 17 and reached 10% (Table 1). As was seen for the other three animals experimentally challenged with MPXV during this study, orthopoxvirus antibodies were detected on day 13 p.i. (Table 1). Viral DNA in blood and oral samples, as well as viral shedding from oral cavity samples occurred on day six (Fig. 5, Table 1). Also as observed in the other transmission groups, the primary challenged animal survived MPXV infection. Animal #8128 was the naive animal housed across from #8132. Viral DNA from oral secretions was detected on day 10; however viral shedding from oral cavity samples did not occur until day 20 as did detection of viral DNA from blood samples (Fig. 5, Table 1) Detectable levels of orthopoxvirus antibodies began on day 24 (Table 1); which was also observed with the naive animal (#9080) in the other respiratory group. On day 24, #8128 had four lesions and approximately 9% weight loss (Table 1); but because of the extreme morbidity of this animal (unresponsive to touch and diarrhea) it was humanely euthanized. Animal #8128's day 24 blood sample (day of euthanasia) was one of the two blood samples collected during this study that yielded viable virus, (Fig. 5).

### Viral Load in Tissues and Blood Chemistry Values

As we have seen in previous studies, all animals that survived MPXV infection in the current study had detectable levels of MPXV DNA in some of the harvested tissues (Figs S1, S2), without the presence of viable virus. The exception to this was a lesion sample from primary challenged animal #8132 (respiratory group 2) and four samples (tongue, brain, skin and lesion) from the primary challenged animal #8134 (bedding group) which was euthanized while still recovering from infection (Fig. S3); all of which yielded viable virus via tissue culture methods. All three moribund secondarily-infected animals that had to be euthanized had high levels of virus in the majority of tissues tested, with animal #8130 having viable virus present in 100% of those tissues harvested. The tongue, spleen, skin, and lesion yielded viable virus from all three of the euthanized animals. A liver sample was only taken from two of the moribund secondarily infected animals during the study, #8130 and #8128 and this tissue was positive for live virus from both of these animals (Fig. S3).

**Figure 6:**
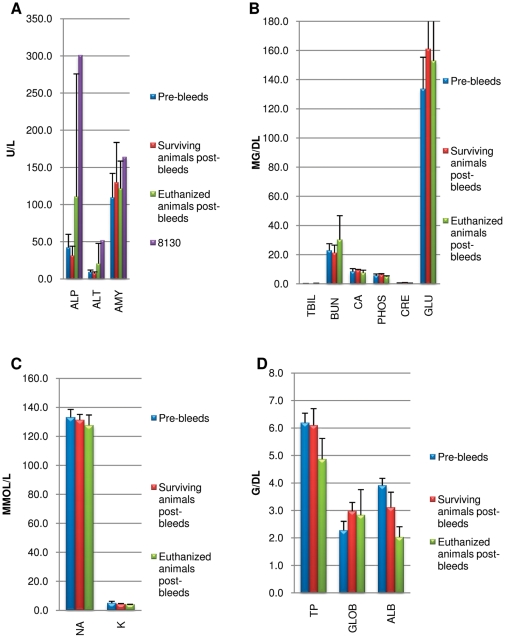
Average blood chemistry values for prairie dogs. Four primary challenged animals were inoculated via intranasal route with 9×10^3^ pfu (0.07XLD_50_) of West African MPXV and placed into one of three experimental groups with naive animals. Blood was taken from each animal before the study began and at time of euthanasia or study completion. Values are for all animals pre-bleeds (n = 14), post-bleeds for animals that survived infection (n = 9) and animals that were euthanized due to extreme morbidity (n = 3); one of which was prairie dog 8130 (A). Error bars show the standard deviation. Values are grouped according to unit of measurement: U/L (A), MG/DL (B), MMOL/L (C) and G/DL (D).

The values for blood chemistries were averaged for three groups: pre-infection for all animals (n = 14); study end for infected animals that survived (n = 9); and at euthanasia for moribund animals (n = 3). When compared, hepatic enzyme values (ALP and ALT) in the euthanized animals were elevated 2–3 times higher than the average value prior to infection, indicating severe hepatocellular necrosis and liver disease (Fig. 6a). In particular, animal #8130 (bedding group naive), which manifest an aggressive disease time course and had to be euthanized on day 15 had abnormal hepatic enzyme levels suggestive of severe liver disease as well as increased blood amylase levels and decreased albumin (ALB) levels indicating potential hepatobiliary disease (Fig. 6a). Also suggestive of liver failure, average ALB values were significantly decreased when comparing the pre-infection values to post values for both surviving and euthanized animals (p = 0.0003 and 0.0045 respectively) (Fig. 6d). Glucose and globulin levels were significantly elevated in surviving animals' post bleed values compared to the pre-infection averages (p = 0.0402 and 0.0004 respectively) (Fig. 6b, 6d). In euthanized animals' blood samples, glucose and globulin levels were also elevated; however no statistical significance was seen when compared to pre-infected values. A trend in elevated BUN levels was observed in euthanized animals' levels compared to pre-infection averages, suggestive of dehydration, which was also clinically noted (Fig. 6b, 6d). No other trends or significant differences were observed in the other blood chemistry values.

## Discussion

In the current study, all four animals experimentally challenged with MPXV developed orthopoxvirus antibodies on day 13 p.i. In the naive animals which were secondarily exposed, we used the date of initial orthopoxvirus antibody production to estimate the date of MPXV infection as a result of disease transmission. Additionally, the four primary challenged animals developed lesions between days 10–13, as did 3/4 challenged animals in a previous study with a similar challenge dose [13]. Another study which utilized a slightly higher inoculation dose, found animals develop lesions at a similar time-frame regardless of the difference in challenge inoculum (days 9–12) [12]. If it is assumed based on antibody response of challenged animals that the development of orthopoxvirus antibodies occurs 13 days after infection with MPXV and that disseminated lesions develop 9–13 days after MPXV infection, the three naive animals in the bedding group were most likely infected with MPXV 0–2 days (#8130) or 5–9 (#s8135 and 8118) days after placement in the trough based on antibody production and initial lesion presentation. Because of the gap in estimated exposure days, it is possible that the first naive animal that became infected subsequently infected the other two secondarily infected animals in this group; but not likely given that animals typically do not shed viable virus until 6–10 days after exposure. As well, measurable infectious virus was not found in animal #8130's oral secretions until day eight. The three naive animals co-housed with an infected animal were most likely infected 7–11 days (# 8136) and 11–15 days (#s8133 and 8154) after the start of the study based on antibody production and lesion presentation. It is noteworthy that samples from the co-housed naive animals were positive for viable virus in oral secretions earlier than detectable levels of viral DNA in blood samples, which is not consistent with what is typically seen during an experimental challenge with MPXV. Additionally, the oral samples from all three naives were positive for viable MPXV as early as day 13, which would suggest these animals were infected between days 3–7 (based on the observation that primary challenged animals begin shedding from the oral cavity 6–10 days after initial infection). However, the primary challenged animal in this group did not begin shedding virus until day 10 p.i., making the likelihood of infection between days 3–7 impossible. The most likely explanation is that the naive animals were either grooming the primary challenged animal or being exposed to the virus through the shared food, water, or living areas. The grooming scenario is most likely as infected animals tend to eat and drink infrequently when they are sick and the co-housed animals were observed to huddle tightly together. Based on antibody production and lesion presentation, the level of virus exposure was not high enough to cause an active infection in these naive animals until days 7–11 or 11–15 p.i. Similar days of infection were estimated for the two naive animals in the respiratory secretion group; between days 11–15 based on initial lesion formation and antibody response. These secondarily infected animals in the respiratory groups had delayed oral cavity viral shedding which matched the time-frame until antibody production and lesion presentation, unlike that seen from the naive animals in the co-housed group. This further supports the theory that the naive animals in the co-housed group were grooming the primary challenged animal or being exposed to virus from food/water sources. Since peak viral shedding from the oral cavity occurs around day 13 from those animals experimentally challenged, as seen in this study and a previous study [13]; the 11–15 day hypothesized timeframe of MPXV exposure would be supported by this data and previous studies.

All four animals that were experimentally challenged with 9×10^3^ pfu of MPXV recovered from infection. However, three of the eight naive, secondarily exposed animals were euthanized due to extreme morbidity including severe diarrhea in all three animals, one animal with too many lesions to count, and one animal that became unresponsive to touch. Several possibilities exist for this observed difference in morbidity and mortality within the secondarily infected animals. One possibility is that the virus that was transmitted from the experimentally challenged animals had a mutation that resulted in increased pathogenicity within the naive animals. Data generated from the 2003 U.S. MPXV outbreak showed that viral isolates from a MPXV infected human and a MPXV infected prairie dog (infected by an unknown African rodent) only had a single nucleotide difference after one or two transmission cycles [8]. Based on this data, it is unlikely that the virus excreted from each of our primary challenged animals mutated resulting in a more virulent strain. It is possible that the naive animals received the virus in a more efficient or natural route than the experimentally challenged animals. However, since there were no bites or scratches on the naive animals, they most likely received the virus via an intranasal or oral route of infection similar to the experimentally challenged animals. The most likely explanation for the increased morbidity and mortality is that the secondarily infected animals were exposed to a higher effective dosage of virus. Based on the observation that these animals shed large quantities of virus from nasal and oral secretions during the current and previous studies with this animal model, this would be a reasonable explanation [12]. Or perhaps a combination of the above differences occurred; the virus was transmitted to the naive animals by both nasal and oral routes resulting in a higher dosage of virus and resulting increased morbidity and mortality.

When comparisons of blood chemistry values was performed, euthanized animals showed increased levels of hepatic enzymes (ALP and ATL) and decrease in ALB suggestive of liver disease. Similar to our findings, a comprehensive-retrospective study of humans infected with MPXV found elevated transaminase and hypoalbuminemia were common abnormalities in patients [16]. Other blood chemistry changes in moribund euthanized animals compared to pre-infection blood levels suggest malnutrition (hypoproteinemia), dehydration (elevated BUN) and stress (hyperglycemia). We can therefore hypothesize that viral infection in these animals may lead to death due to organ failure (especially liver), malnutrition, and dehydration. Although only two blood samples were positive for viable virus; subsequent DNase treatment of two PCR positive blood samples that did not yield viable virus suggested that these samples did contain intact virions (data not shown). This suggests that low levels of intact virions are present in some of the viral DNA positive blood samples, however the levels are below the limit of detection with the tissue culture methods utilized.

Previous studies have shown that prairie dogs shed large quantities of MPXV from oral cavity, nasal and ocular secretions as well as feces [12], and with this study we are able to demonstrate that these animals could transmit the virus to naive animals through exposure to contaminated bedding, direct contact, and respiratory secretions and/or nasal mucous. All of our secondarily exposed animals (n = 8) were infected with MPXV during the course of the study. It is possible that differences in animal behavior is partly responsible for the 100% transmission we observed in the current study, especially in the co-housed group in which animals tend to huddle tightly together and possibly groom one another. Additionally, because the cages used for the respiratory secretion group were not separated by physical barrier, the question did arise as to whether these animals were actually able to touch noses through the holes in the cages and therefore exchange nasal discharge. If this scenario did occur, this would not be a true test of respiratory transmission but possibly a combination of respiratory transmission and/or nasal mucous secretion transmission. Interestingly, some of the animals secondarily exposed appeared to manifest more severe disease (severe diarrhea in three animals, one animal with too many lesions to count, and one animal that became unresponsive to touch) resulting in euthanasia of three of these animals. These findings are supportive of the hypothesis that transmission to the naive animals involved a higher viral dose and/or different/additional route of infection than the experimentally challenged animals. Although disease severity within some of the naive animals appeared greater compared to the primary challenged animals, the disease courses were very similar in both groups including inappetence leading to weight loss, development of disseminated lesions, production of orthopoxvirus antibodies and shedding of similar levels or in some instances higher levels of virus from oral cavity secretions. The ability of the prairie dog MPXV model to transmit the West African strain of MPXV animal-to-animal confirms observations made during the 2003 US outbreak [5], [6]. During outbreaks of MPXV in human populations, the West African strain has not been documented as being transmitted human-to-human [9], [10]. Although this animal model may not recapitulate what we believe to know about interhuman transmission of the West African clade of MPXV, it confirms observations that the prairie dog is an efficient MPXV transmitting host and an ideal animal model to study orthopoxvirus infections.

Although other animal models have been utilized for the study of MPXV (reviewed by [17]), our studies have shown that the prairie dog is an excellent model of human systemic orthopoxvirus disease with a prolonged incubation period, generalized lesions, as well as observable differences between the two viral clades [12], [13], similar to human monkeypox disease course. Through the current study we have added to the benefits of this animal model by demonstrating the ability to study animal-to-animal transmission. These animals were able to readily transmit the West African strain of MPXV by three routes, including natural respiratory secretions and/or nasal mucous exchange, similar to what we believe to occur in human-to-human MPXV and variola virus transmission. We can use these studies to extrapolate to possible human exposure and make recommendation for healthcare workers and those people who live in areas where MPXV is endemic: fomite exposure is similar to healthcare workers handling infected bedding or bandages from sick patients; direct contact is similar to families living in a house together; and respiratory secretion groups mimic possible human exposure to MPXV from a sneeze or cough from an infected individual in close proximity. Future studies with this model could be very useful in evaluating infection control measures during outbreaks, testing of anti-viral therapies to diminish transmission rates, as well as helping to understand differences in rates of transmission between the two strains of MPXV within human populations.

## Materials and Methods

### Ethics statement

All animals were handled in strict accordance with good animal practice as defined by the relevant national and/or local animal welfare bodies, and all animal work was approved by the CDC Institutional Animal Care and Use Committee (IACUC) under an approved protocol (2115-211DAMPRAC) issued by CDC IACUC specifically for this study.

### Animals

Wild-caught, juvenile black-tailed prairie dogs (*Cynomys ludovicianus*) were obtained from Berthoud Colorado. The animals involved were purchased from a vendor which utilized humane live-trapping techniques (wire cage traps) to capture free-living healthy young animals (<1year old). Only animals free from any signs of illness (as determined by a veterinarian) were transported to the Centers for Disease Control and Prevention (CDC) in Atlanta, GA. Once the animals arrived at the CDC, they were quarantined and housed appropriately under an approved CDC IACUC protocol (1718CARPRAC) until the start of the study. At time of the start of this study animals were approximately 20 months old and had been prescreened by a veterinarian and determined to be in good health status and found negative for the presence of anti-orthopoxvirus antibodies. 14 animals were used in this study. The average starting weight for primary challenged animals was 932 grams (range 904–982), and the average for naive animals was 994 grams (range 584–1265). A sterile PIT tag was injected subcutaneously at the base of the neck for animal identification and non-invasive recording of body temperature.

### Transmission strategies

All animals were housed in an animal Biological Safety Level-3 (ABSL-3) animal room. Animals were divided into three experimental groups to evaluate routes of transmission: 1) bedding/fomite, 2) co-housed, or 3) respiratory. For the bedding/fomite group, one animal was challenged with virus and housed for 16 days in a large trough (approximately 2.5 feet wide by 5 feet long) with a dust-free moisture absorbent pellet made from recycled newspapers (Paperchip regular texture pellets, Shepherd Specialty Papers). After day six the bedding was not changed due to findings from previous studies in which shedding from excrement most often began on day six [12], [13]. Previous prairie dog MPXV studies have demonstrated that peak viral shedding occurs between days 9–13. Therefore, the challenged animal was left in the trough until day 16 at which time it was removed and humanely euthanized. Three naive animals were placed into the large trough for housing with the contaminated bedding and this bedding was not changed for 39 days. For the co-housed group, one animal was challenged with virus and housed in a large trough (approximately 2.5 feet wide by 5 feet long) with 3 naive animals for 38 days; bedding was changed weekly. Finally, for the respiratory secretion group; four large metal rabbit cages with holes on one side (3 sq. feet cage area; 16.438″ high×19.125″ wide×25.875″ long) were utilized to individually house four animals (two challenged, 2 naive). Cages were placed approximately one inch apart with ventilation holes (1 inch in diameter) from the challenged animals' cages facing the holed-side from the naive animals' cages within a Duo-Flow biosafety cabinet with negative directional airflow, in a similar design as illustrated in another study [18]. Primary challenged animals were monitored for the subsequent 31 days while the naive animals were monitored for 38 days. Additionally, two phosphate-buffered saline (PBS) infected negative control animals were housed individually in large (12.13″×23.38″×209.00″) rat cages with aerosol filter tops for 38 days. PBS animals, bedding/fomite group animals, and the respiratory secretion grouped animals were kept in two Duo-Flow biosafety cabinets with negative directional airflow to allow air to be HEPA filtered before leaving the unit. The trough containing the co-housed group of animals was in the ABSL-3 animal room. Animals were cared for in accordance with CDC Institutional Animal Care and Use Committee (IACUC) guidelines under an approved protocol (2115-211DAMPRAC). In addition to prairie dog chow and water, animals were provided with monkey biscuits and mixed nuts for added dietary enrichment.

### Viruses

The West African MPXV strain (MPXV-USA-2003-044), isolated during the 2003 U.S. outbreak [6], [8], [DQ011153], was used in this study. The virus underwent two passages in African green monkey kidney cells (BSC-40) prior to seed pool production; preparations used for animal challenge inoculums were purified via a sucrose cushion.

### Animal inoculation

The challenge dose (9×10^3^ pfu) was calculated based on the morbidity and mortality rates observed in the authors' previous studies. Briefly, a challenge dose of 6×10^3^ pfu resulted in 25% mortality and disease morbidity included rash lesions and viral shedding identified in oral cavity samples in 100% of animals [12], [13]. Stocks of virus were diluted in PBS. Inocula titers were immediately re-confirmed, post- challenge, by standard plaque assay (as described below). Animals were infected via an intranasal (i.n.) route of inoculation while under general anesthesia using 3–5% isoflurane administered through a veterinary vaporizer. Animals were inoculated with a total volume of 10 ul i.n. (5 ul in each nostril). Additionally, 2 animals were mock infected with PBS.

### Observations and sampling

Post-inoculation, individual animals were observed daily for signs of morbidity, or malaise (inappetence, decreased activity, recumbancy with reluctance to move, etc.) and clinical lesions or rash for the study duration. Two times a week animals were anesthetized with isoflurane for sampling purposes. Samples and measurements collected included an oral swab (area swabbed included inner cheeks, tongue and the hard palate); blood, weight, temperature and lesion count (if applicable). Strict euthanasia criteria were adhered to throughout the study as follows: any animal that became unresponsive to touch, lost 25% or more starting body weight, or accrued a total score of 10 on the following scale was humanely euthanized: decreased activity (2 points); lethargy, unsteady gait, inappetence (3 points each); labored breathing and recumbency (5 points each).

### Necropsy and tissue specimen collection

Necropsies on all animals were performed according to CDC IACUC-approved standards in an ABSL-3 laboratory and utilizing full ABSL-3 PPE. Samples taken during necropsy included: submandibular lymph nodes/salivary glands, spleen, lung, liver (for two animals), tongue, skin, lesion (if present), brain, oral swab, and blood. Instruments were decontaminated with 5% Microchem and 70% ethanol and allowed to dry between collections of each tissue. Tissue samples were frozen at −70°C prior to further processing. Oral samples were collected with sterile individual swabs and stored frozen without diluent. After animals were sampled each day, serum was immediately separated from whole blood and stored at −20°C to be processed for serology and clinical chemistry levels (see below). Tissues and samples were subsequently processed and further prepared for DNA analysis, and virus isolation (see below).

### Sample preparation for PCR and viral growth

Sample processing was performed under BSL-2 conditions with BSL-3 work practices. For whole blood samples, 100 ul of EDTA treated blood was used for DNA extraction and the remaining untreated blood was used for tissue culture propagation. 400 ul of sterile PBS was added to each swab collected. The swab extraction tube systems (SETS) (Roche) protocol was used to recover sample from the swab. DNA was extracted from 100 ul of the swab lysate. The remaining swab eluate was used for virus isolation. For tissue preparation, 1 ml aliquots of PBS and SPEX bead (SPEX Sample Prep) were prepared. The PBS/bead aliquot was then poured into a 1 ml tube containing the individually weighed tissue sample. The GenoGrinder 2000 (SPEX Sample Prep) was then used following the manufacturer's instructions to create a tissue homogenate. 100 ul of the homogenate was then used for DNA extraction. The remaining homogenate was used for virus isolation. The BioRobot EZ-1 Workstation (Qiagen) was used for DNA extraction from all blood, swab and tissue samples using the Tissue Kit. Samples were incubated at 55°C in lysis buffer for an hour to inactivate viable virus particles prior to DNA extraction.

### Real-time PCR analysis

Samples were tested by real-time PCR using forward and reverse primers and probes complimentary to the conserved *Orthopoxvirus* (OPXV) E9L (DNA polymerase) gene [19]. MPXV DNA (10 fg–1 ng) was used as positive controls. A positive sample produced CT values (in duplicate) of 37 or below. A weakly positive sample displayed CT values 38–39 (duplicates).

To determine whether a subset of PCR positive EDTA blood samples contained intact virions, a DNase assay (Li et al. personal communication) was used. In brief, the assay allows for the determination of how much of the viral DNA was protected from DNase treatment as a measure of how much of the DNA was incorporated within intact virions.

### Virus-tissue infectivity

All samples were stored at −70°C until virus isolation was attempted. Previous analyses demonstrated that real-time PCR detection of MPXV DNA is an assay which can detect trace amounts of MPXV DNA in samples which do not contain viable virus [5]. Therefore, specimens were first tested for presence of OPXV DNA by PCR and, if positive, were subsequently evaluated for viable virus by tissue culture propagation. Each positive sample was titrated in duplicate using 10 fold dilutions of swab eluate, whole blood or tissue slurry on BSC-40 cell monolayers, incubated at 35.5°C and 6% CO_2_ for 72 hours, and subsequently stained with crystal violet and 10% formalin to visualize plaques. Titers were expressed as pfu per milliliter (pfu/ml) of blood or swab eluate; or pfu per gram (pfu/g) of tissues.

### Serologic analysis

Serum was separated from whole blood and transferred to a clean tube and stored at −20C prior to analysis. A modified ELISA was used for analysis of anti-OPXV immunoglobulin types A and G in separated serum as previously described in detail [12].

### Blood chemistry values

Serum was separated from whole blood and transferred to a clean tube and stored at −20C prior to analysis. The Piccolo blood chemistry analyzer (Abaxis) was utilized to determine the following blood chemistry profiles: sodium (NA), potassium (K), phosphorus (PHOS), glucose (GLU), calcium (CA), blood urea nitrogen (BUN), creatinine (CRE), alkaline phosphatase (ALP), alanine aminotransferase (ALT), amylase (AMY), total bilirubin (TBIL), albumin (ALB), total protein (TP) and globulin (GLOB).

### Statistical analyses

A paired t-test was utilized to compare chemistry values and a p-value of ≤0.05 was considered statistically significant.

## Supporting Information

Figure S1 **Detection of viral DNA within necropsy samples harvested from the bedding group and co-housed group of animals.** Four primary challenged animals were inoculated via intranasal route with 9×10^3^ pfu (0.07XLD_50_) of West African MPXV and placed into one of three experimental groups with naive animals. Animals that were euthanized due to extreme morbidity are indicated by crosses. At time of death or at study completion, full necropsies were completed and tissues were tested for viral DNA (fg/ul) and viable virus (pfu/g) in a log scale. Results for animals within the bedding group (A) and co-housed group (B) are shown.
(TIF)

Figure S2 **Detection of viral DNA within necropsy samples harvested from the respiratory groups of animals.** Four primary challenged animals were inoculated via intranasal route with 9×10^3^ pfu (0.07XLD_50_) of West African MPXV and placed into one of three experimental groups with naive animals. Animals that were euthanized due to extreme morbidity are indicated by crosses. At time of death or at study completion, full necropsies were completed and tissues were tested for viral DNA (fg/ul) and viable virus (pfu/g) in a log scale. Results for animals within respiratory group 1 (A) and respiratory group 2 (B) are shown.
(TIF)

Figure S3 **Levels of viable virus within necropsy tissues.** Four primary challenged animals were inoculated via intranasal route with 9×10^3^ pfu (0.07XLD_50_) of West African MPXV and placed into one of three experimental groups with naive animals. Three of the naive animals (indicated by crosses) were euthanized due to extreme morbidity during the study. At time of death or at study completion, full necropsies were completed and tissues were tested for viral DNA (fg/ul) and viable virus (pfu/g) on a log scale. Only those animals testing positive for viable virus are shown.
(TIF)
