# Supplementary figures and images for: Monkeypox Disease Transmission in an Experimental Setting: Prairie Dog Animal Model
Source: PLoS One. 2011 Dec 2;6(12):e28295. doi: 10.1371/journal.pone.0028295 (PMC3229555; doi:10.1371/journal.pone.0028295)

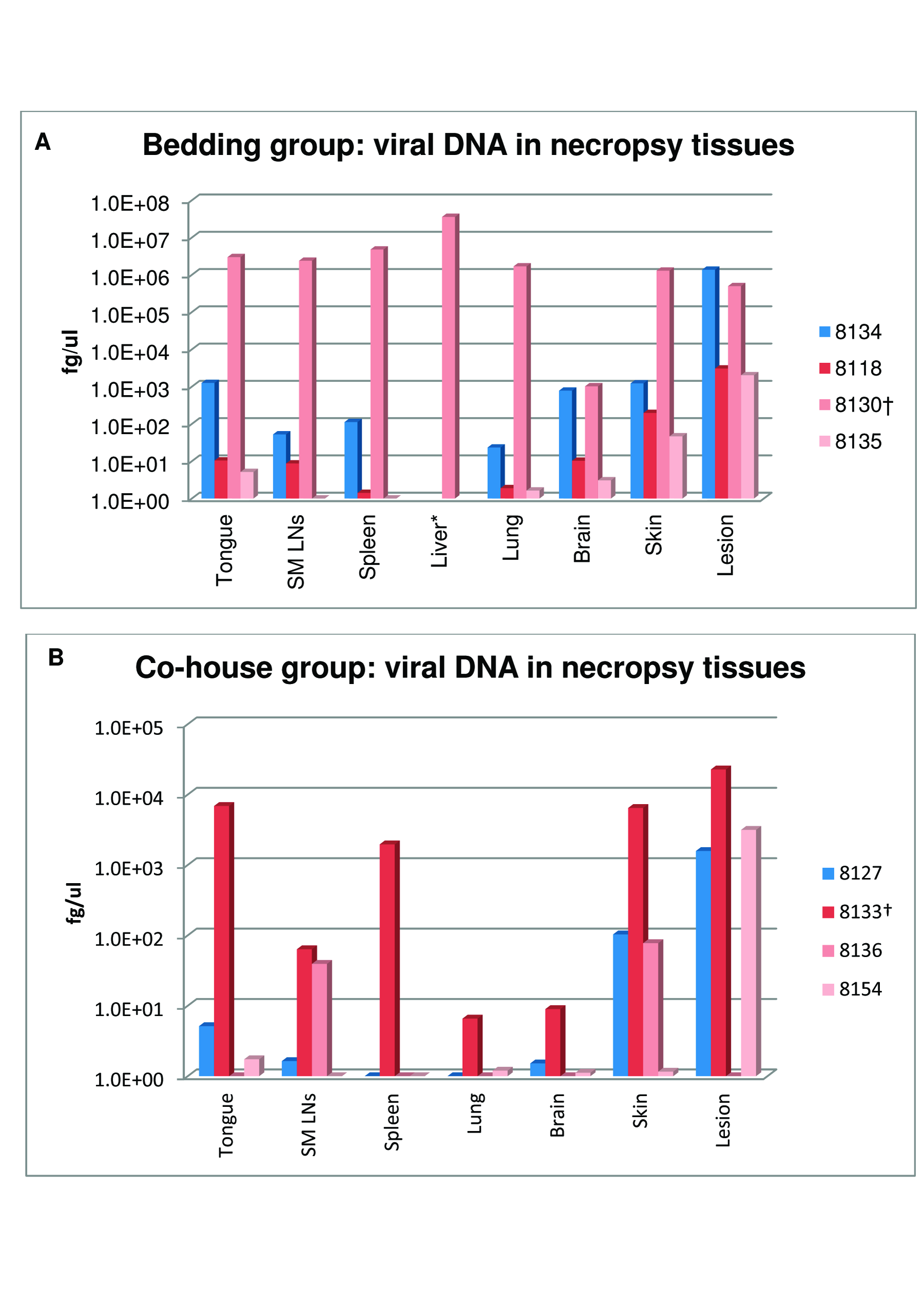

Supplement: Figure S1 — Detection of viral DNA within necropsy samples harvested from the bedding group and co-housed group of animals. Four primary challenged animals were inoculated via intranasal route with 9×10^3^ pfu (0.07XLD50) of West African MPXV and placed into one of three experimental groups with naive animals. Animals that were euthanized due to extreme morbidity are indicated by crosses. At time of death or at study completion, full necropsies were completed and tissues were tested for viral DNA (fg/ul) and viable virus (pfu/g) in a log scale. Results for animals within the bedding group (A) and co-housed group (B) are shown. (TIF) [file pone.0028295.s001.tif]

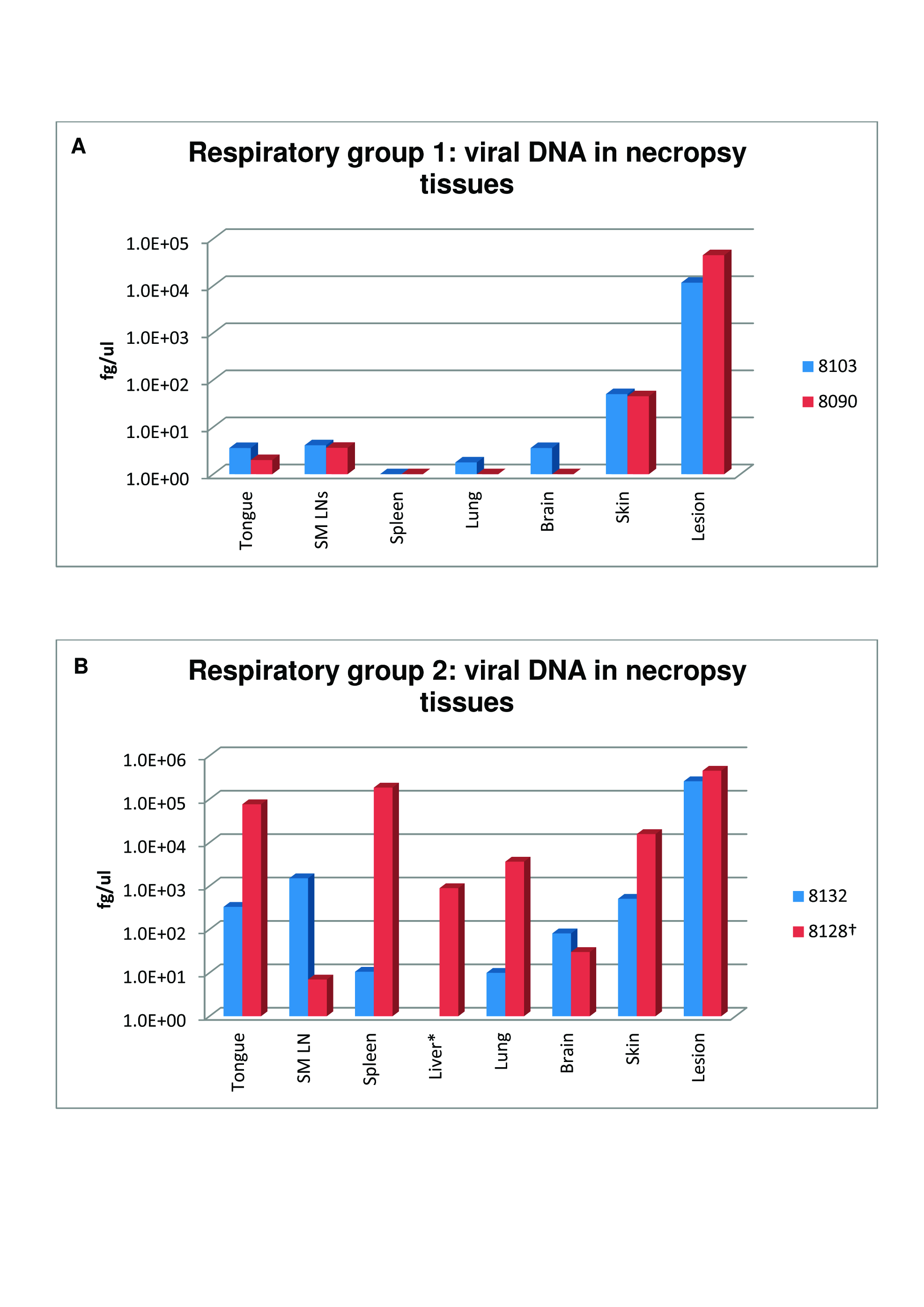

Supplement: Figure S2 — Detection of viral DNA within necropsy samples harvested from the respiratory groups of animals. Four primary challenged animals were inoculated via intranasal route with 9×10^3^ pfu (0.07XLD50) of West African MPXV and placed into one of three experimental groups with naive animals. Animals that were euthanized due to extreme morbidity are indicated by crosses. At time of death or at study completion, full necropsies were completed and tissues were tested for viral DNA (fg/ul) and viable virus (pfu/g) in a log scale. Results for animals within respiratory group 1 (A) and respiratory group 2 (B) are shown. (TIF) [file pone.0028295.s002.tif]

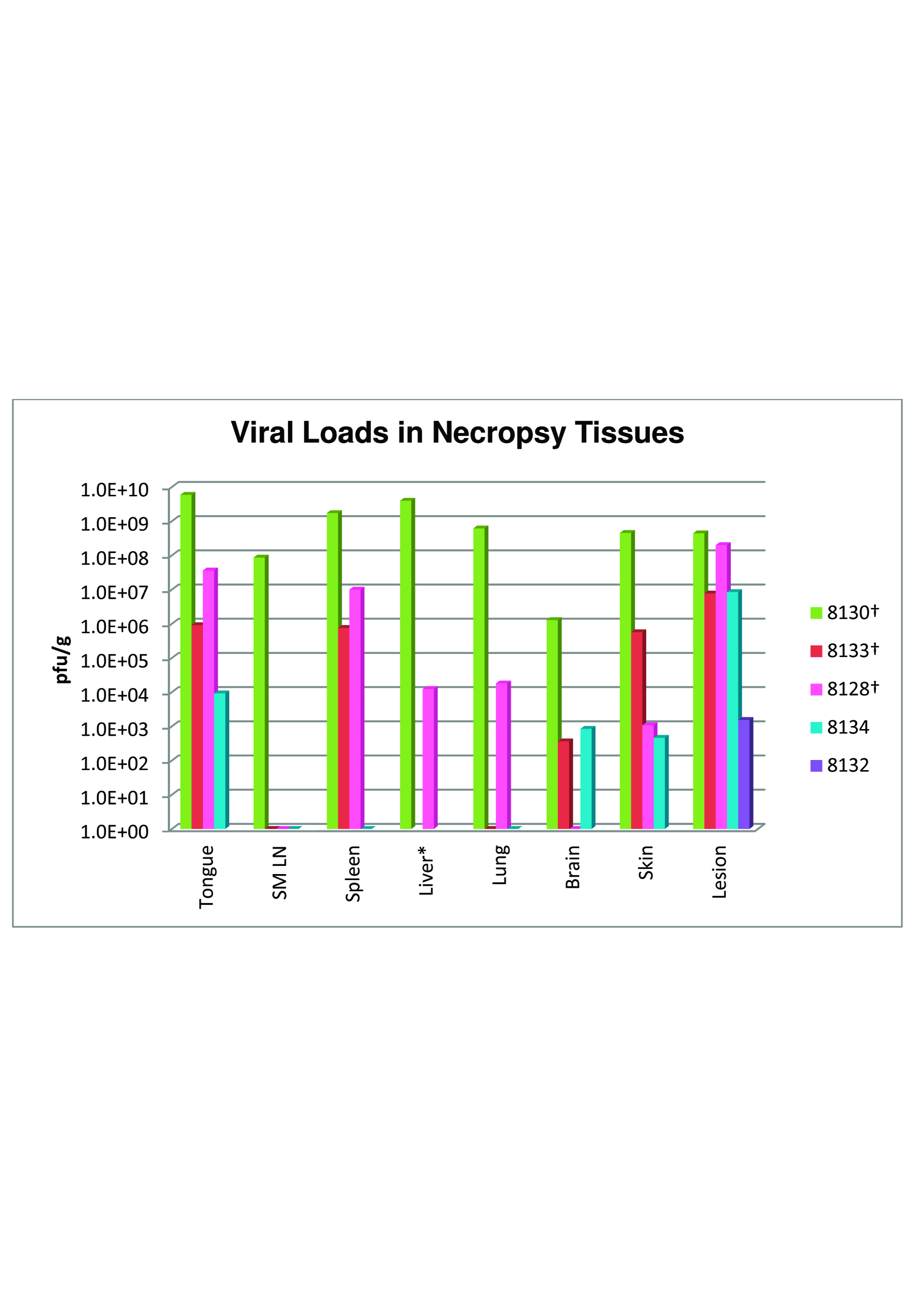

Supplement: Figure S3 — Levels of viable virus within necropsy tissues. Four primary challenged animals were inoculated via intranasal route with 9×10^3^ pfu (0.07XLD50) of West African MPXV and placed into one of three experimental groups with naive animals. Three of the naive animals (indicated by crosses) were euthanized due to extreme morbidity during the study. At time of death or at study completion, full necropsies were completed and tissues were tested for viral DNA (fg/ul) and viable virus (pfu/g) on a log scale. Only those animals testing positive for viable virus are shown. (TIF) [file pone.0028295.s003.tif]
